# 5‐HT_2B_ receptor antagonists attenuate myofibroblast differentiation and subsequent fibrotic responses in vitro and in vivo

**DOI:** 10.14814/phy2.12873

**Published:** 2016-08-01

**Authors:** Anna Löfdahl, Kristina Rydell‐Törmänen, Catharina Müller, C. Martina Holst, Lena Thiman, Gunilla Ekström, Christina Wenglén, Anna‐Karin Larsson‐Callerfelt, Gunilla Westergren‐Thorsson

**Affiliations:** ^1^ Lung BiologyDepartment of Experimental Medical ScienceLund UniversityLundSweden; ^2^ AnaMar ABLundSweden

**Keywords:** 5‐HT, bleomycin, extracellular matrix, fibroblast, pulmonary fibrosis, *α*‐SMA

## Abstract

Pulmonary fibrosis is characterized by excessive accumulation of connective tissue, along with activated extracellular matrix (ECM)‐producing cells, myofibroblasts. The pathological mechanisms are not well known, however serotonin (5‐HT) and 5‐HT class 2 (5‐HT_2_) receptors have been associated with fibrosis. The aim of the present study was to investigate the role of 5‐HT_2B_ receptors in fibrosis, using small molecular 5‐HT_2B_ receptor antagonists EXT5 and EXT9, with slightly different receptor affinity. Myofibroblast differentiation [production of alpha‐smooth muscle actin (*α*‐SMA)] and ECM synthesis were quantified in vitro, and the effects of the receptor antagonists were evaluated. Pulmonary fibrosis was also modeled in mice by subcutaneous bleomycin administrations (under light isoflurane anesthesia), and the effects of receptor antagonists on tissue density, collagen‐producing cells, myofibroblasts and decorin expression were investigated. In addition, cytokine expression was analyzed in serum. Lung fibroblasts displayed an increased *α*‐SMA (*P* < 0.05) and total proteoglycan production (*P* < 0.01) when cultured with TGF‐*β*1 together with 5‐HT, which were significantly reduced with both receptor antagonists. Following treatment with EXT5 or EXT9, tissue density, expression of decorin, number of collagen‐producing cells, and myofibroblasts were significantly decreased in vivo compared to bleomycin‐treated mice. Receptor antagonization also significantly reduced systemic levels of TNF‐*α* and IL‐1*β*, indicating a role in systemic inflammation. In conclusion, 5‐HT_2B_ receptor antagonists have potential to prevent myofibroblast differentiation, in vitro and in vivo, with subsequent effect on matrix deposition. The attenuating effects of 5‐HT_2B_ receptor antagonists on fibrotic tissue remodeling suggest these receptors as novel targets for the treatment of pulmonary fibrosis.

## Introduction

Pulmonary fibrosis is characterized by an excessive accumulation and remodeling of connective tissue and is considered to be the result of an imbalanced wound healing response, which fails to terminate correctly (Wilson and Wynn 2009). Progressive pulmonary fibrosis is often detected late, when tissue remodeling is extensive, creating a challenging approach for effective treatment (Meltzer and Noble 2008). To date there are no curative therapies for pulmonary fibrosis, leaving lung transplantation as a last resort.

The accumulation and deposition of extracellular matrix (ECM) in fibrosis are thought to rely on myofibroblasts, a cell‐type featuring increased expression of alpha‐smooth muscle actin (*α*‐SMA) and enhanced production of ECM proteins such as collagens and proteoglycans (Lijnen and Petrov 2002; Venkatesan et al. 2002). Myofibroblast differentiation is induced by profibrotic growth factors, and especially by transforming growth factor (TGF)‐*β*1 (Verrecchia and Mauviel 2007). Another profibrotic mediator is serotonin (5‐hydroxytryptamine [5‐HT]), and studies support 5‐HT‐driven production of other profibrotic mediators and cell differentiation (Gairhe et al. 2012; Chen et al. 2014). The physiological levels of 5‐HT are normally low due to uptake from plasma into platelets (Mercado and Kilic 2010). However, upon tissue damage, for example, endothelial injury, platelets are activated and released 5‐HT, which results in increased local concentrations of 5‐HT (Schattner 2014; Mauler et al. 2015). An alternative source of pulmonary 5‐HT originates from pulmonary neuroendocrine cells (Pan et al. 2006) and activated mast cells (Kushnir‐Sukhov et al. 2007; Larsson‐Callerfelt et al. 2013a).

5‐HT mediates effects through the 5‐HT receptors, and the 5‐HT class 2 (5‐HT_2_) receptors, especially subtypes 5‐HT_2A_ and 5‐HT_2B_, have been associated with chronic fibrosis (Konigshoff et al. 2010; Dees et al. 2011). Lungs of patients with idiopathic pulmonary fibrosis (IPF) present increased expression of 5‐HT_2A_ and 5‐HT_2B_ receptors, with strong 5‐HT_2B_ receptor localizations in densely fibrotic areas, so‐called fibroblastic foci (Fabre et al. 2008; Konigshoff et al. 2010). Furthermore, studies investigating 5‐HT_2A_ and 5‐HT_2B_ receptor antagonism in vivo have displayed promising antifibrotic effects (Fabre et al. 2008; Konigshoff et al. 2010). However, despite several descriptions of a correlation between 5‐HT and fibrosis, no study has yet suggested a mechanism for the interaction.

The differentiation into myofibroblasts and subsequent ECM synthesis appear to be key events in the development of pulmonary fibrosis, and thus represent a highly interesting therapeutic target (Hinz 2007). We therefore hypothesized that 5‐HT through interaction with 5‐HT_2B_ receptors participate in remodeling processes observed in pulmonary fibrosis, by exerting effect on myofibroblast differentiation. This study aimed to investigate potential antifibrotic effects of two small molecular 5‐HT_2B_ receptor antagonists with slightly different receptor affinity, EXT5 and EXT9. The antifibrotic effects of the antagonists were studied both in vitro and in vivo. Our results suggest involvement of 5‐HT_2B_ receptors in myofibroblast differentiation and ECM production in fibrosis, emphasizing the need for further investigations of 5‐HT_2B_ receptor antagonists as potential therapeutics for pulmonary fibrosis.

## Material and Methods

### Receptor binding and functionality assay of the 5‐HT_2B_ receptor antagonists

5‐HT_2_ receptor binding and functionality profiles of the 5‐HT_2B_ receptor antagonists EXT5 (332.8 g/mol) and EXT9 (279.2 g/mol) (AnaMar AB, Lund, Sweden) [Patent PCT Int. Appl. (2008), WO 2008071980 A1] were evaluated in vitro by Eurofins Panlabs (Taipei, Taiwan), using Chinese hamster ovary cells (CHO‐K1) that stably express human recombinant 5‐HT_2A_, 5‐HT_2B_, or 5‐HT_2C_ receptors. Binding profiles were evaluated with radio ligand‐binding assay (in vitro assays 271650, 271700, and 271800). 5‐HT_2A_ and 5‐HT_2B_ receptor function for the two antagonists were measured with homogeneous time‐resolved fluorescence (HTRF) quantification of IP_1_ accumulation in CHO‐K1 (in vitro assays 355250 and 355260). Compounds creating a ≥ 50% inhibition of 5‐HT (5 nmol/L) induced fluorescence response specified receptor antagonist activity. 5‐HT_2C_ receptor functionality was assessed with [^35^S] GTP*γ*S for quantification of bound GTP*γ*S. Compounds creating a ≥ 50% inhibition of 5‐HT (0.1 *μ*mol/L) induced [^35^S] GTP*γ*S binding response specified receptor antagonist activity (in vitro assay 355200). The receptor antagonists were screened at 0.003, 0.03, 0.3, 3, and 30 *μ*mol/L. A nonlinear, least square regression analysis was used to determine half maximal inhibitory concentration (IC_50_) values.

### Cell culture and cell stimuli

Human lung fibroblasts (HFL‐1, CCL‐153, ATCC, Manassas, VA, USA) were expanded in Eagle's minimal essential medium (MEM, Biochrom, Berlin, Germany) supplemented with 1% penicillin–streptomycin (PEST), 1% glutamine, and 10% fetal clone serum (FCIII, Thermo Scientific, Waltham, MA) at 37°C, 5% CO_2_. The cells were used in passages 14–20 with the majority of experiments performed in passages 18–20. Equivalent supplemented Dulbecco's modified Eagle's medium (DMEM, Sigma‐Aldrich, St Louis, MO) with 0.4% FCIII was used in general experimental conditions at 37°C, 10% CO_2_, unless otherwise stated.

HFL‐1 cells were stimulated with 5‐HT (1 or 10 *μ*mol/L, 5‐HT hydrochloride, Tocris, Bristol, UK) alone or in combination with TGF‐*β*1 (10 ng/ml) (R&D systems, Minneapolis, MN). TGF‐*β*1, known as a general inducer of remodeling (Westergren‐Thorsson et al. 2010), was added together with 5‐HT to stimulate fibroblast differentiation, mimicking physiologic remodeling. Fibroblasts were treated with the 5‐HT_2B_ receptor antagonists [dissolved in DMSO (Sigma‐Aldrich)] EXT5 (1 and 10 *μ*mol/L) or EXT9 (1, 5, and 10 *μ*mol/L) in combination with TGF‐*β*1 and 5‐HT (1 *μ*mol/L), investigating potentially inhibitory effects of the antagonists.

### Immunocytochemistry (ICC) of *α*‐SMA, 5‐HT_2A_, and the 5‐HT_2B_ receptor

HFL‐1 plated on glass chamber slides and cultured for 24 h with TGF‐*β*1 (10 ng/mL) ± 5‐HT (1 *μ*mol/L or 10 *μ*mol/L) or medium alone, were fixed in 4% formaldehyde solution. Rehydrated cells were used for ICC visualization of *α*‐SMA and 5‐HT_2A_ and 5‐HT_2B_ receptor expression in HFL‐1, according to a standard protocol. Briefly, cells were incubated with the following primary antibodies: *α*‐SMA (1:2000, C6198, Sigma‐Aldrich), 5‐HT_2A_ (1 *μ*g/mL, sc‐166775, Santa Cruz, Dallas, TX), 5‐HT_2B_ (2.5 *μ*g/mL, AP01188PU‐N, Acris Antibodies GmbH, Herford, Germany), or 5‐HT_2C_ (1:500, PA5‐27164, Thermo Scientific) for 1.5 h room temperature (RT), rinsed in TBS, before incubation with secondary antibody for 45 min (RT), and mounting. For *α*‐SMA, cells were permeabilized with 0.2% Triton X‐100 (5 min) before ICC. In all protocols DAPI was used for nuclear staining.

### Western blot analysis of *α*‐SMA in human lung fibroblasts

HFL‐1, densely seeded in six‐well cell culture plates, were stimulated with either TGF‐*β*1 (10 ng/mL), 5‐HT (1 *μ*mol/L or 10 *μ*mol/L), or the combination of TGF‐*β*1 and 5‐HT, with or without the 5‐HT_2B_ receptor antagonists EXT5 (10 *μ*mol/L) or EXT9 (10 *μ*mol/L). In addition, a preventive regimen (pretreatment with the receptor antagonists 1 h before adding the combination of TGF‐*β*1 and 5‐HT) was investigated. After 24 h, cell lysates were collected in supplemented 1% NP‐40 (Sigma‐Aldrich) and total protein content in cell lysate was quantified with BCA protein assay kit (Thermo Scientific). Collected lysates were then reduced, and size separated protein samples were transferred to a PVDF membrane (Merck Millipore, Darmstadt, Germany). The membrane was incubated for 2 h with *α*‐SMA antibody (0.3–1 *μ*g/mL, ab5694, Abcam, Cambridge, UK), along with *β*‐tubulin antibody (1:30,000–40,000, ab6046, Abcam) or GAPDH antibody (0.2 *μ*g/mL, sc‐47724, Santa Cruz) in blocking buffer (0.5% casein in 0.5% TBS‐Tween), before washing in TBS‐Tween, and incubation (30 min, RT) with a secondary DyLight680 or DyLight800‐conjugated antibody, prior to final washing steps. Core protein bands of *α*‐SMA and endogenous controls (GAPDH and *β*‐tubulin) were imaged with Odyssey FC (LI‐COR Inc., Lincoln, NE), controlled and analyzed by Image Studio v.3.1 (LI‐COR Inc.). Data are presented as fold change of *α*‐SMA compared to control (medium alone or the combination of TGF‐*β*1 and 5‐HT).

### Measurements of proteoglycan synthesis in human lung fibroblasts

Confluent HFL‐1 cells were subjected to a stepwise culturing regimen. Cells were first cultured in 0.4% FCIII supplemented DMEM for 2 h and then stimulated with TGF‐*β*1 (10 ng/mL), 5‐HT (1 *μ*mol/L), or the combination of TGF‐*β*1 and 5‐HT with or without the receptor antagonists EXT5 (10 *μ*mol/L) and EXT9 (10 *μ*mol/L) in 0.4% FCIII supplemented sulfate‐poor medium (Invitrogen, Waltham, MA). After 2 h, [^35^S] (50 *μ*Ci/mL) was added to the wells. Cell medium and lysate were then collected after 24‐h stimulation. Total protein content was quantified with BCA protein assay kit (Thermo Scientific), and proteoglycans were extracted by column‐based separation and size separated with gel electrophoresis as described previously (Nihlberg et al. 2010). Bands of sized separated [^35^S] sulfate‐labeled proteoglycans were quantitated for the proteoglycans decorin, versican, perlecan, and biglycan with densitometry. The gels were imaged with Molecular Imager FX (Bio‐Rad, Laboratories Inc., Hercules, CA) and analyzed with Quantitative One 4.6. The amount of proteoglycans was related to total protein content (mg), and results were compared to controls (medium alone or the combination TGF‐*β*1 and 5‐HT).

### Bleomycin‐induced pulmonary fibrosis in vivo model

Female C57/Bl6 mice, aged 12.5 w (Scanbur research A/S, Karlslunde, Denmark), were injected subcutaneously three times/week for 2 weeks with bleomycin 50 IE/animal (Baxter Medical AB, Kista, Sweden) as described previously (Rydell‐Tormanen et al. 2012). Treatment groups (seven mice/group) received daily per oral (p.o.) administration of either EXT5 (30 mg/kg) or EXT9 (30 mg/kg) dissolved in water‐based Tween80 (2.5 w/v %, Sigma‐Aldrich) in parallel to the bleomycin administrations. Positive controls received bleomycin only and negative controls received saline injections, with p.o. administration of vehicle. Following sacrifice, 14 days poststudy initiation, lungs were fixed in 4% formaldehyde and embedded in paraffin. Blood was collected from subclavian arteries and serum stored at −20°C. Study protocols were approved by the local ethics committee (Malmö/Lund, Sweden, M103‐14).

### Immunofluorescence

For all analysis, 4‐*μ*m rehydrated sections and a standard protocol were used. Briefly, sections were rehydrated, pretreated with proteinase K (20 mg/mL) for 30 min (37°C), and washed in TBS. A primary antibody was applied and the sections incubated for 1.5 h (RT), before rinsing in TBS. A secondary antibody (standard dilution 1:200) was applied and incubated for 45 min (RT). The sections were then rinsed (for double staining, new primary and secondary antibodies were applied according to the protocol) and mounted. The following primary antibodies were used: *α*‐SMA (1:2000, C6198, Sigma‐Aldrich), prolyl‐4‐hydroxylase (P4H, 1:200, CTX101468, GeneTex, Irvine, CA), or decorin (1:200, BAF1060, R&D Systems), nuclei were visualized with DAPI and for negative controls primary antibodies were omitted. All quantifications were done in a blinded fashion.

#### Myofibroblasts

Myofibroblasts (defined as solitary cells copositive for *α*‐SMA and P4H) were visualized by double staining and the number of double‐positive cells were manually counted in five randomly obtained images of lung parenchyma (20 ×  magnification), and results were given as number of double‐positive cells/mm^2^.

#### Collagen‐producing cells

The number of single‐positive cells for P4H was used as a measurement of ongoing fibrosis (as P4H is necessary in collagen synthesis); the number of P4H single‐positive cells, negative for *α*‐SMA, was manually counted in five randomly obtained images of lung parenchyma (20 ×  magnification), and results were given as number of single‐positive cells/mm^2^.

#### Decorin

Tissue area positive for decorin was analyzed according to a previously described protocol (Rydell‐Tormanen et al. 2014). The positively stained area was calculated using ImageJ, and the results were given as positively labeled area per image (0.14 mm^2^).

### Histology

Rehydrated tissue sections (4 *μ*m) (5–7 animals from each treatment group) were stained with hematoxylin/eosin and Masson's trichrome.

#### Tissue density

Tissue sections were stained with hematoxylin/eosin according to standard protocol and the fraction of tissue of the total lung parenchymal area (%) was analyzed in 7–10 randomly obtained images (20 ×  magnification) per animal using ImageJ (Wayne Rasband, NIH, Bethesda, MD).

#### Collagen staining

Tissue sections were stained with Masson's trichrome (HT15, Sigma‐Aldrich) according to manufacturer's protocol, dehydrated, and mounted in pertex. Whole tissue sections were scanned (20 ×  magnification) using Olympus (Hamburg, Germany) VS120 slide scanner with XV image processer L100 VS‐ASW. With Visiopharm (VIS 6.1.0, Hoersholm, Denmark), parenchymal tissue were manually determined and analyzed for collagen content. Larger airways, vessels, and pleura were excluded from regions of interest. Positive‐stained area for collagen (blue staining) were quantified and related to total tissue area (excluding airspaces). Results were given as positive‐labeled area of collagen versus total tissue area (%). Image viewer software VS‐OlyVIA (version 2.9) (Olympus Soft Imaging solutions GmbH; Münster, Germany) was used for image visualization.

### Cytokine assay of serum samples

Serum samples from negative (saline) and positive (bleomycin) controls as well as treatment groups (EXT5, 30 mg/kg or EXT9, 30 mg/kg) were run in a MSD Multispot assay (K15048G‐1, Meso Discovery, Rockville, MD) according to manufacturer's instructions. Mediators were analyzed with a MSD Sector for the measurement of interleukin (IL)‐1*β*, IL‐2, IL‐4, IL‐5, IL‐6, IL‐10, IL‐12p70, keratinocyte chemoattractant/growth‐regulated oncogene (KC‐GRO) or interferon gamma (INF*γ*) and tumor necrosis factor alpha (TNF‐*α*). Images and data were processed using the software 2400 MSD Discovery Workbench version 4.0.

### Statistical analysis

Data were statistically tested using Graph Pad Prism 5.04 (in vitro data, La Jolla, CA) and Analyse‐It v.2.26 (in vivo data, Leeds, UK). The following statistical analyses were performed: one sample *t*‐test (*P* value two tailed) for in vitro analyses and Kruskal–Wallis test with LSD post hoc test for in vivo analyses. Significant *P* ‐values are defined as *P ≤ 0.05, **P ≤ 0.01, ***P ≤ 0.005, ****P ≤ 0.001.

## Results

### Profiles of receptor antagonists and 5‐HT_2A_ and 5‐HT_2B_ receptor expression in HFL‐1

**Figure 1:**
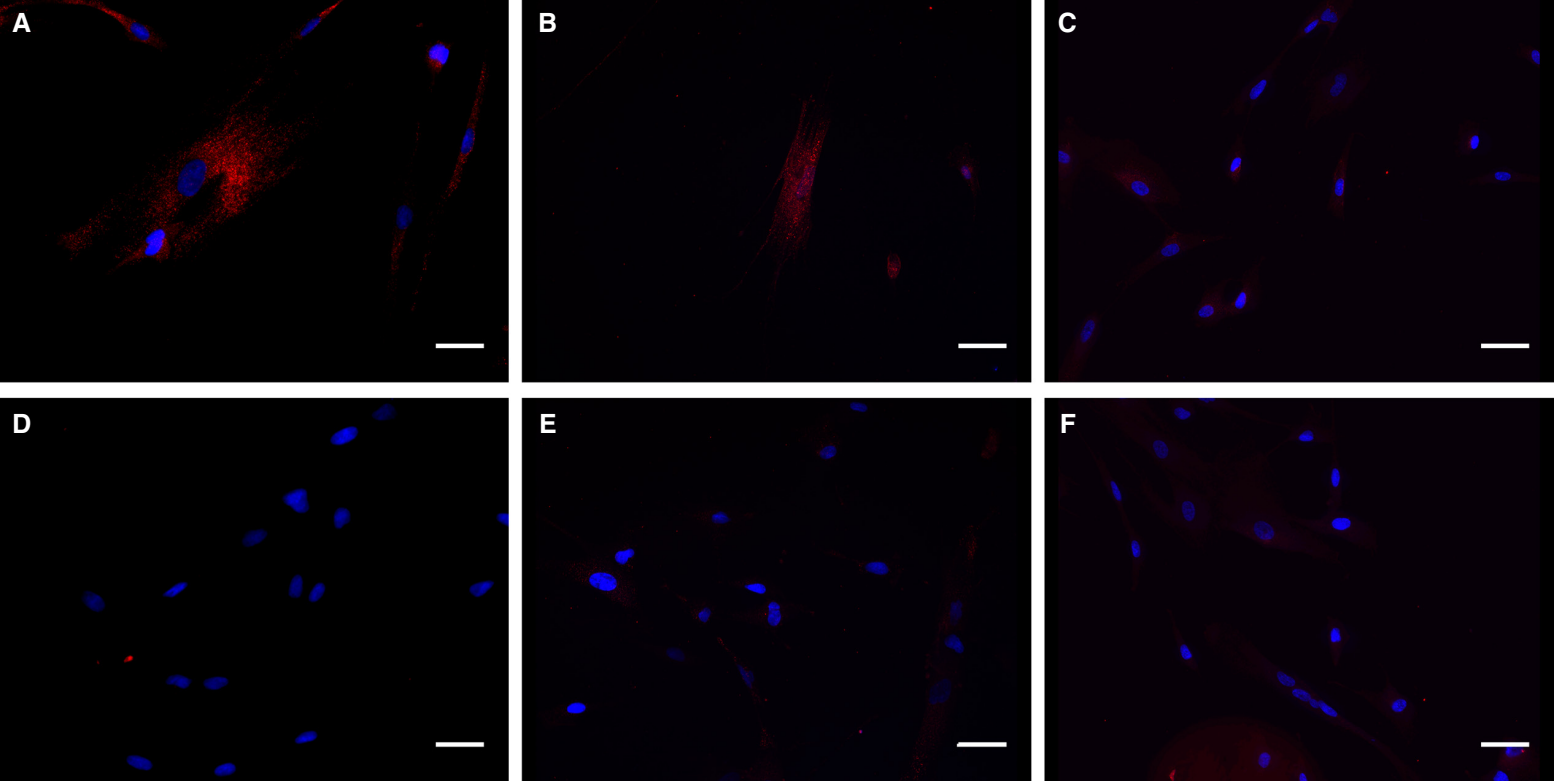
HFL‐1 expression of 5‐HT_2_ receptors. Identification of 5‐HT_2A_ (A), 5‐HT_2B_ (B), and 5‐HT_2C_ (C) receptors on cultured HFL‐1 cells. Isotype‐matched negative controls for 5‐HT_2A_ (D), 5‐HT_2B_ (E), and 5‐HT_2C_ (F) receptor antibodies showed no positive receptor staining. Anti‐5‐HT_2_ receptor antibodies (red), cell nuclear staining with DAPI (blue). Scale bars represent 40 *μ*m.

The 5‐HT_2B_ receptor antagonists EXT5 and EXT9 are structurally well‐defined benzylidene aminoguanidine derivatives that fulfills Lipinski's rule of five (Leeson 2012). Receptor studies of EXT5 and EXT9 indicated that the antagonists not only primarily antagonized 5‐HT_2B_ receptors, but also presented low to moderate affinity to the 5‐HT_2A_ and 5‐HT_2C_ receptors. Furthermore, the two antagonists possessed slightly different selectivity to the 5‐HT_2_ receptors (Table 1). Our experiments in vitro utilizing 1 *μ*mol/L of 5‐HT, necessitated an antagonist concentration of 10 *μ*mol/L to effectively compete for 5‐HT_2B_ receptor occupancy, since 5‐HT is a potent agonist, demonstrating an half maximal effective concentration (EC_50_) of approximately 2.4 nmol/L (Eurofins Panlabs Inc., 2016). The expression of 5‐HT_2_ receptors on HFL‐1 was identified with antibody labeling, confirming the expression of both 5‐HT_2A_ and 5‐HT_2B_ receptors, but not 5‐HT_2C_ by this cell type (Fig. 1).

**Table 1:** Receptor binding and functionality profiles of EXT5 and EXT9

Compound	K_i_			IC_50_		
	5‐HT_2A_	5‐HT_2B_	5‐HT_2C_	5‐HT_2A_	5‐HT_2B_	5‐HT_2C_
EXT5 (*μ*mol/L)	1.01	0.045	0.31	7.54	0.082	5.11
EXT9 (*μ*mol/L)	0.46	0.026	0.11	0.84	0.029	0.65

K_i_, inhibition constant; IC_50_, half maximal inhibitory concentration.

### 5‐HT_2B_ receptor antagonists reduced myofibroblast differentiation in vitro

**Figure 2:**
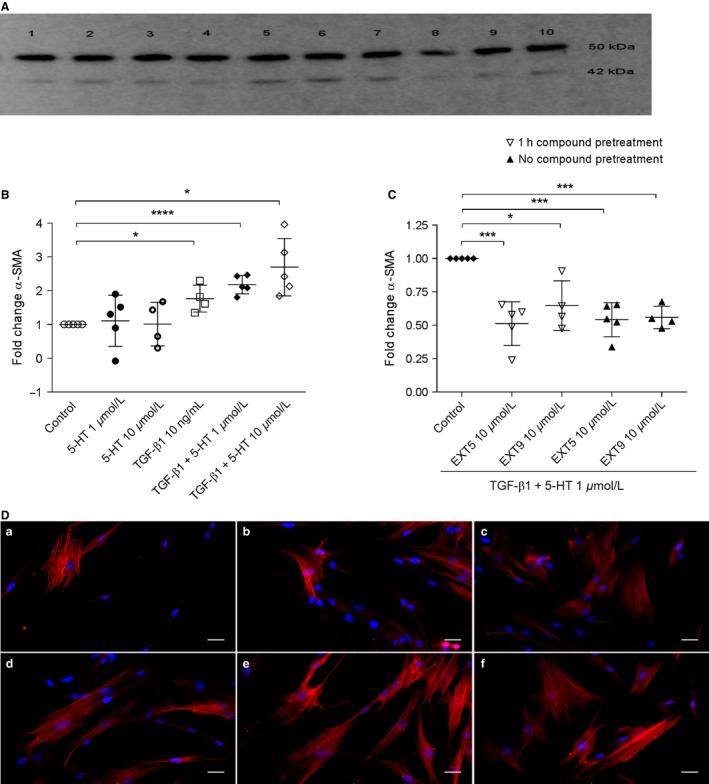
*α*‐SMA production in HFL‐1 treated with TGF‐*β*1 + 5‐HT and 5‐HT_2B_ receptor antagonists. Core protein bands of *β*‐tubulin (upper row, 50 kDa) and *α*‐SMA (lower row, 42 kDa) were visualized with western blot from cell lysates collected from HFL‐1 treated with EXT5 (1 h pretreatment) (1), EXT9 (1 h pretreatment) (2), 5‐HT (1 *μ*mol/L) (3), 5‐HT (10 *μ*mol/L) (4), TGF‐*β*1 (10 ng/mL) (5), TGF‐*β*1 (10 ng/mL) + 5‐HT (1 *μ*mol/L) (6), TGF‐*β*1 (10 ng/mL) + 5‐HT (10 *μ*mol/L) (7), medium (8), EXT5 (without pretreatment) (9), or EXT9 (without pretreatment) (10) for 24 h. The image is representative for five conducted experiments (A). The relative quantity of intracellular *α*‐SMA (B) was quantified and normalized to control (cells without added stimuli) after 24 h. TGF‐*β*1 as well as the combination of TGF‐*β*1 and 5‐HT significantly increased the amount of *α*‐SMA in cells. This increase induced by TGF‐*β*1 and 5‐HT (control) (C) was abolished by the addition of 5‐HT_2B_ receptors antagonists EXT5 (10 *μ*mol/L) and EXT9 (10 *μ*mol/L). The one sample *t*‐test (*P* value two tailed) was used; *P ≤ 0.05, ***P ≤ 0.005, ****P ≤ 0.001 compared to control, *n* = 4–5 (*n* = number of experiments). Illustrative images of *α*‐SMA‐positive labeling (D), visualized by ICC in HFL‐1 cells cultured without any added stimuli (*a*) or stimulated with TGF‐*β*1 (10 ng/mL; *b*), 5‐HT (1 *μ*mol/L; *c*), 5‐HT (10 *μ*mol/L; *d*), TGF‐*β*1 (10 ng/mL) and 5‐HT (1 *μ*mol/L; *e*), or costimulation with TGF‐*β*1 (10 ng/mL) and (5‐HT 10 *μ*mol/L; *f*). *α*‐SMA is visible as red intracellular labeling, and nuclei were visualized by DAPI (blue), scale bars represent 30 *μ*m.

Myofibroblast differentiation was evaluated with western blot quantification of *α*‐SMA (Fig. 2A) in HFL‐1 cells stimulated with TGF‐*β*1, 5‐HT, or the combination of TGF‐*β*1 and 5‐HT (Fig. 2B). Stimulation with TGF‐*β*1 (10 ng/mL) alone and costimulation with TGF‐*β*1 and 5‐HT (1 or 10 *μ*mol/L) significantly (*P* < 0.05) increased *α*‐SMA protein expression in HFL‐1, whereas 5‐HT alone did not increase *α*‐SMA compared to nonstimulated cells. A strong tendency toward an additive effect of 5‐HT (10 *μ*mol/L) was found when administered together with TGF‐*β*1 compared to TGF‐*β*1 alone (*P* = 0.053, Fig. 2B). Furthermore, the effect of the 5‐HT_2B_ receptor antagonists was investigated in cells costimulated with TGF‐*β*1 and 5‐HT. Treatment with the 5‐HT_2B_ receptor antagonists EXT5 (10 *μ*mol/L) or EXT9 (10 *μ*mol/L) in cells costimulated with TGF‐*β*1 and 5‐HT (1 *μ*mol/L) significantly reduced the levels of *α*‐SMA (*P* < 0.005) compared to cells costimulated only with TGF‐*β*1 and 5‐HT (1 *μ*mol/L) (Fig. 2C). Preventative antagonist administration (1 h pretreatment) showed no additional inhibitory effects (Fig. 2C). In addition, the presence of *α*‐SMA was visualized in HFL‐1 cells by immunocytochemistry (Fig. 2D).

### 5‐HT_2B_ receptor antagonists decreased proteoglycan production in lung fibroblasts

**Figure 3:**
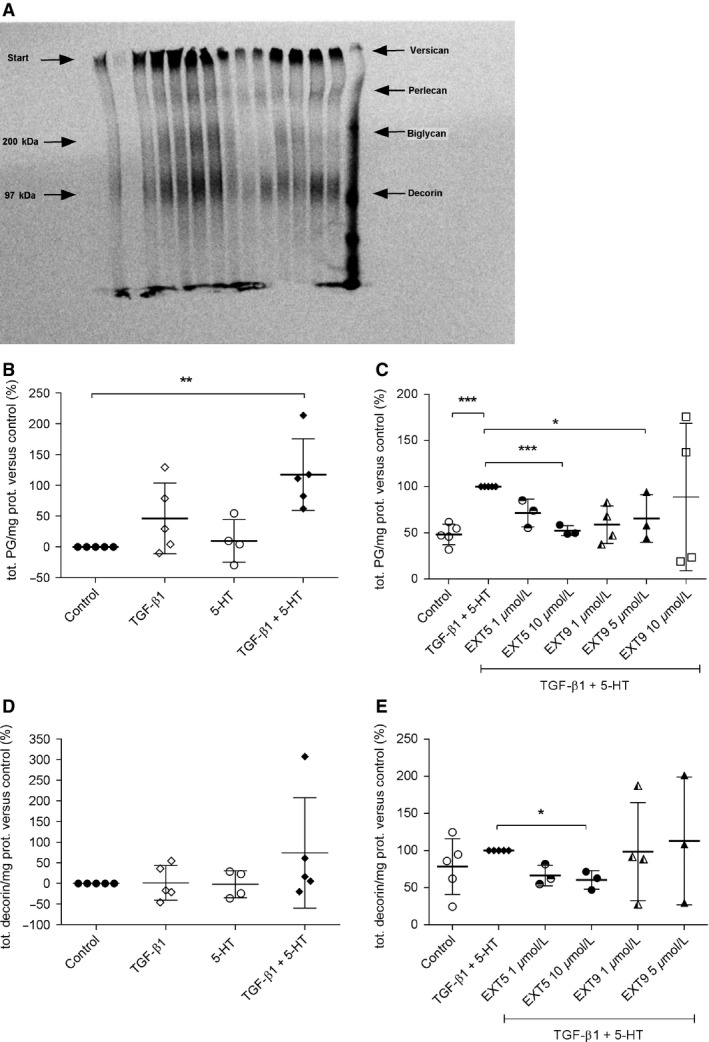
Effects of 5‐HT_2B_ receptor antagonists on proteoglycan synthesis. Bands of sized separated [^35^S] sulfate‐labeled proteoglycans, extracted from HFL‐1 medium, were exemplified in one representative image, with identified areas of separated proteoglycans; versican, perlecan, biglycan, and decorin (A). The relative amount of newly synthesized sulfate [^35^S]‐labeled proteoglycans from HFL‐1 cells stimulated with TGF‐*β*1 (10 ng/mL), 5‐HT (1 *μ*mol/L), TGF‐*β*1 (10 ng/mL) + 5‐HT (1 *μ*mol/L), or cultured in medium alone (control) was analyzed with densitometry, normalized to total protein content, and compared to control (B) The combination of 5‐HT (1 *μ*mol/L) and TGF‐*β*1 induced significantly increased total proteoglycan production that was inhibited by the addition of 5‐HT_2B_ receptor antagonists EXT5 (10 *μ*mol/L) and EXT9 (1 *μ*mol/L) (control: TGF‐*β*1 + 5‐HT [1 *μ*mol/L]) (C). Synthesis of the proteoglycan decorin following incubation with TGF‐*β*1 (10 ng/mL), 5‐HT, or TGF‐*β*1 and 5‐HT was quantified, normalized to total protein content, and compared to cells incubated with only medium (control) (D). No increased production of decorin was found in response to any treatment, but a tendency toward increased production was detected following stimulation with a combination of TGF‐*β*1 and 5‐HT. Treatment with the 5‐HT_2B_ receptor antagonist EXT5 (10 *μ*mol/L) decreased the decorin production significantly (E) compared to cells costimulated with TGF‐*β*1 and 5‐HT (control). For statistical analysis, the one sample *t*‐test (*P* value two tailed) was used; *P ≤ 0.05, **P ≤ 0.01, ***P ≤ 0.005 versus control, *n* = 3–5 (*n* = number of experiments).

Total proteoglycan production was not affected by either TGF‐*β*1 (10 ng/mL) or 5‐HT (1 *μ*mol/L) alone, whereas the combination significantly (*P* < 0.01) increased total proteoglycan production compared to nonstimulated controls (Fig. 3A). The addition of 5‐HT_2B_ receptor antagonists EXT5 (10 *μ*mol/L) or EXT9 (5 *μ*mol/L) to cells costimulated with TGF‐*β*1 and 5‐HT (1 *μ*mol/L) significantly (*P* < 0.05) reduced the total production of proteoglycans (Fig. 3B).

The effect of the antagonists on individual proteoglycans (decorin, biglycan, perlecan, and versican) revealed that EXT5 (10 *μ*mol/L) significantly reduced decorin synthesis (*P* = 0.0317, Fig. 3C and D), whereas there were no significant effects of EXT5 on the production of either perlecan, versican, or biglycan. EXT9 had no significant effects on individual proteoglycans (data not shown).

### 5‐HT_2B_ receptor antagonists attenuates bleomycin‐induced pulmonary remodeling

**Figure 4:**
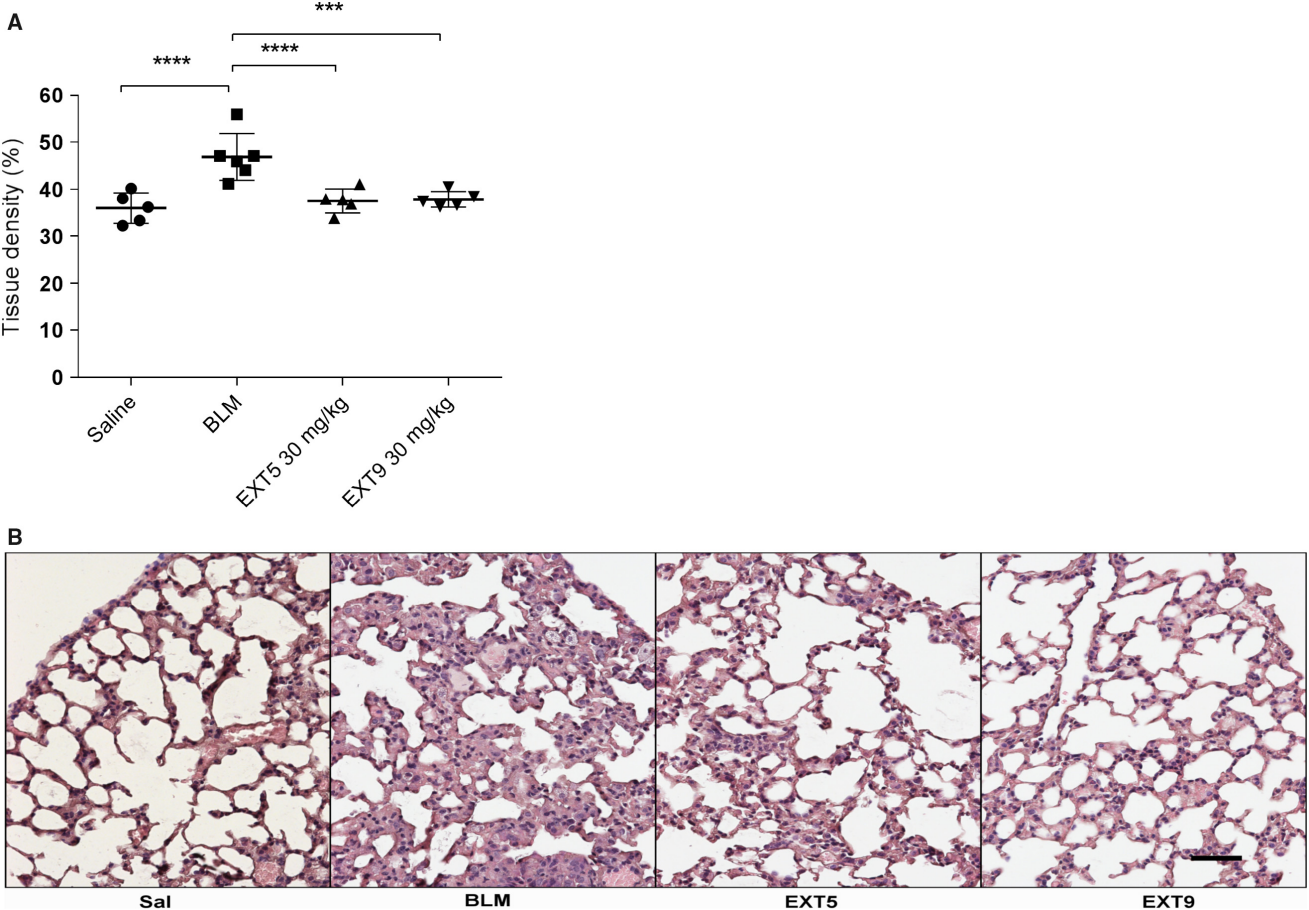
5‐HT_2B_ receptor antagonists decreased bleomycin‐induced pulmonary fibrosis. The lung density, that is, fraction tissue within a lung section, was significantly increased following bleomycin (BLM) administrations, and this increase was completely abolished by the administration of the 5‐HT_2B_ receptor antagonists, EXT5 (30 mg/kg) and EXT9 (30 mg/kg) (A). Illustrative images (B) of 4‐*μ*m thick hematoxylin/eosin‐stained tissue sections from a control animal (Sal) and following BLM administration (BLM), as well as after treatment with EXT5 and EXT9 (30mg/kg). Scale bars represent 50 *μ*m and applicable to all images. Data are presented as individual animals (mean ± SD,* n* = 5–7), the Kruskal–Wallis test with LSD post hoc test was used for statistical analysis; *P ≤ 0.05, **P ≤ 0.01, ***P ≤ 0.005, ****P ≤ 0.001.

Development of pulmonary fibrosis in response to subcutaneous administration of bleomycin was confirmed by significantly increased tissue density of the alveolar parenchyma in animals exposed to bleomycin compared to animals receiving saline injections (*P* < 0.001, Fig. 4A and B). The increased tissue density was completely abolished (*P* < 0.005) by daily administration of the 5‐HT_2B_ receptor antagonists EXT5 (30 mg/kg) or EXT9 (30 mg/kg) (Fig. 4A).

**Figure 5:**
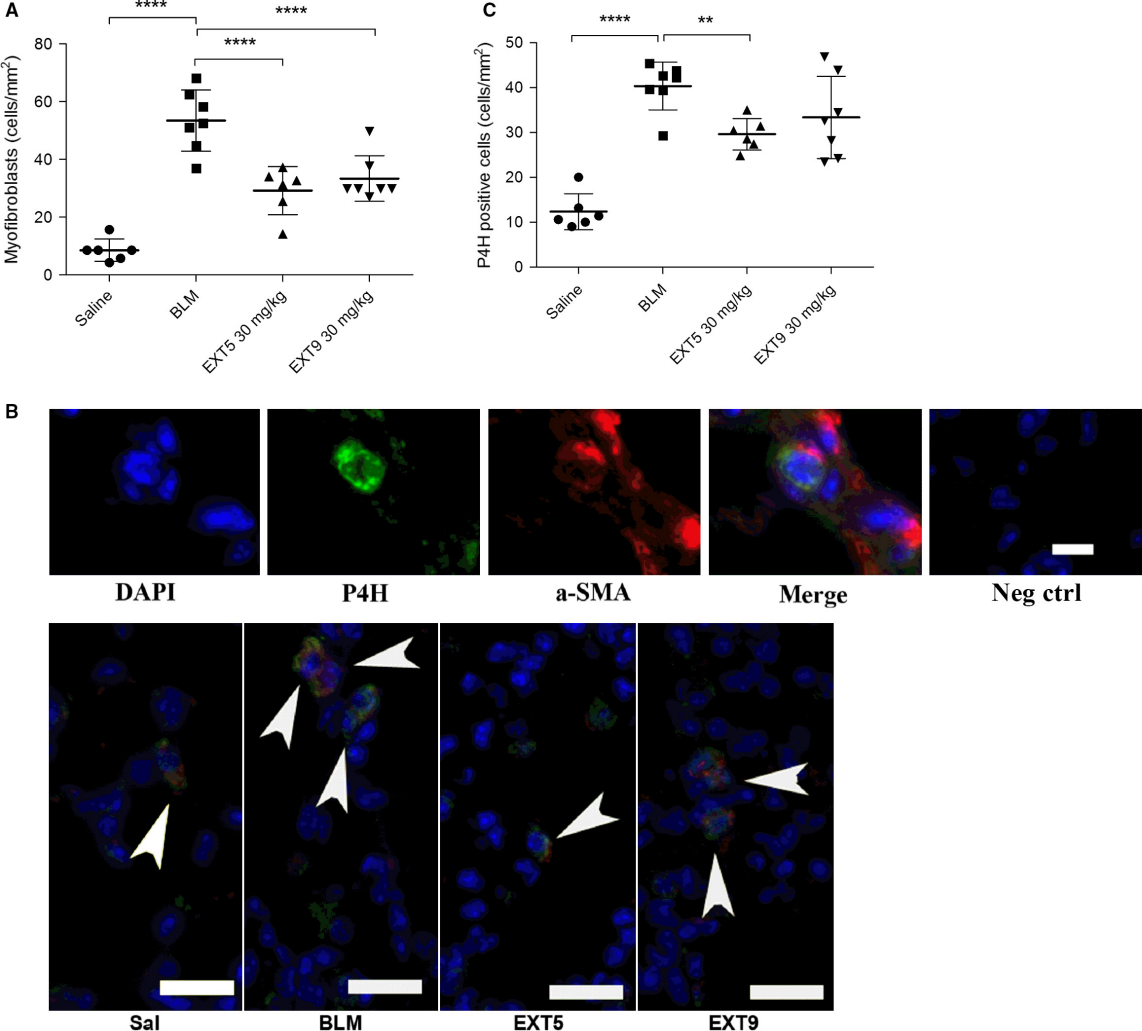
Treatment with 5‐HT_2B_ receptor antagonists decreased the number of myofibroblasts and collagen‐producing cells. Quantification of myofibroblasts (A) and collagen‐producing cells (C) showed that bleomycin (BLM) significantly increased the number of both myofibroblasts and collagen‐producing cells compared to animals receiving only saline administrations. Administration of both 5‐HT_2B_ receptor antagonists (EXT5 [30 mg/kg] and EXT9 [30 mg/kg]) significantly decreased the number of myofibroblasts compared to BLM administration, whereas only EXT5 inhibited the BLM‐induced increased number of collagen‐producing cells. Data are presented as individual animals (mean ± SD,* n* = 5–7) and for statistical analysis, the Kruskal–Wallis test with LSD post hoc test was used; *P ≤ 0.05, **P ≤ 0.01, ***P ≤ 0.005, ****P ≤ 0.001. Descriptive images visualizing myofibroblasts (arrowheads) were defined as cells copositive for *α*‐SMA (red) and prolyl‐4 hydroxylase (P4H, green) (B), whereas collagen‐producing cells were single positive for P4H. Nuclei were visualized by DAPI, and negative controls (omitting the primary antibody) showed no labeling. Scale bars represent 10 *μ*m (upper image) and 20 *μ*m (lower image).

The number of myofibroblasts increased significantly from 9 ± 4 to 53 ± 11 cells/mm^2^ (*P* < 0.0001, Fig. 5A) following bleomycin administration compared to animals receiving saline. Daily administration with EXT5 or EXT9 significantly (*P* < 0.001) reduced the number of myofibroblasts (29 ± 8 cells/mm^2^ and 33 ± 8 cells/mm^2^, respectively) compared to animals receiving bleomycin. Myofibroblasts were visible as solitary cells copositive for *α*‐SMA and P4H, within the lung parenchyma (Fig. 5B). Bleomycin administration resulted in increased collagen synthesis, detected as an increased number of P4H single‐positive cells, compared to animals receiving saline injections (Fig. 5C). Daily administration of EXT5 significantly reduced the bleomycin‐induced increase of P4H‐positive cells (*P* = 0.0091), whereas EXT9 had no effect (*P* = 0.06, Fig. 5C). P4H‐positive cells were identified as solitary, single‐positive cells in the lung parenchyma.

**Figure 6:**
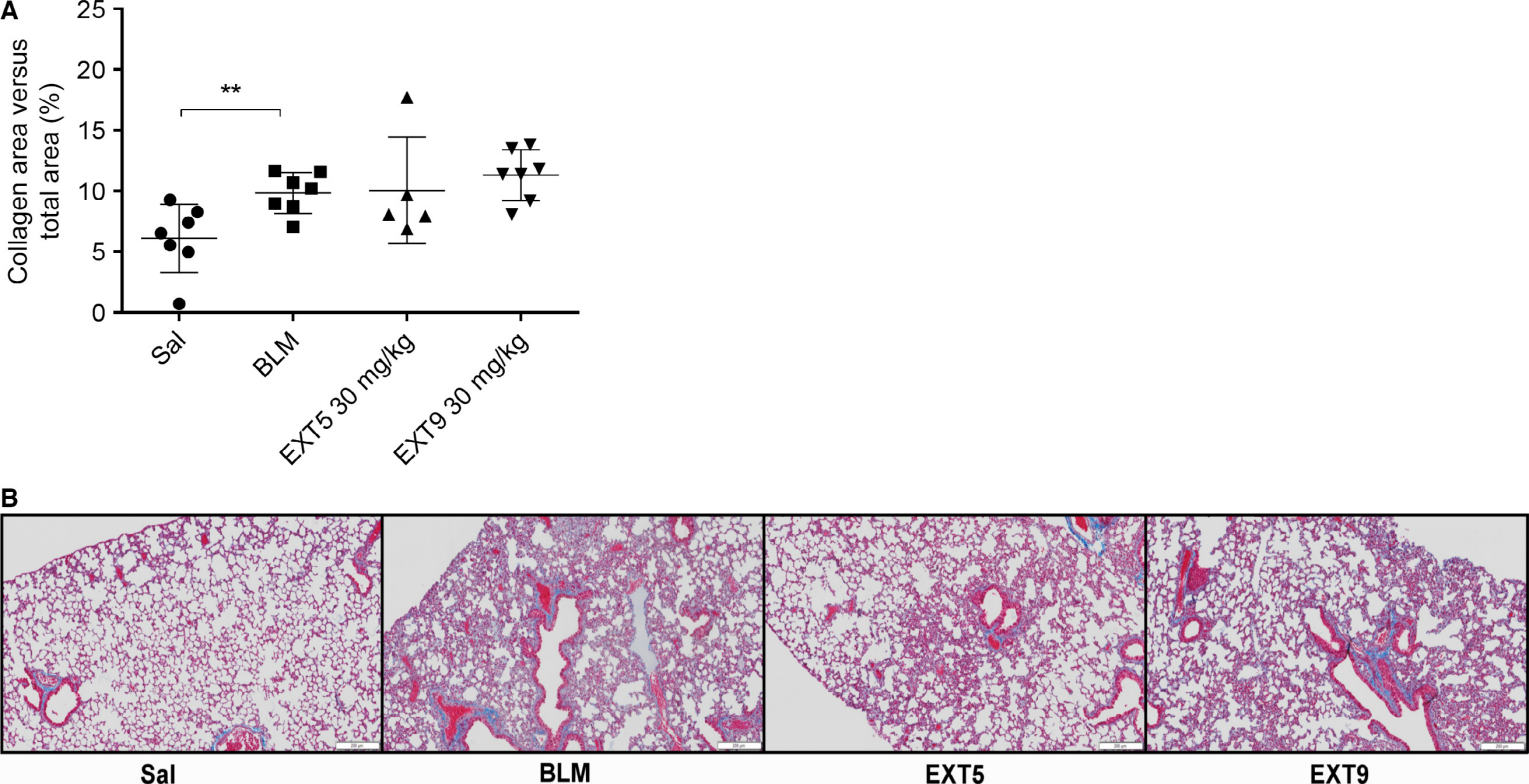
Collagen content in parenchymal lung tissue was increased in bleomycin‐treated mice. Total collagen content related to total examined area was quantified in tissue slides stained for Masson's trichrome. Bleomycin (BLM) administrations increased collagen content in lung parenchyma compared to saline (Sal) (A). Treatment with EXT5 or EXT9 (30 mg/kg) did not affect the amount of collagen compared to BLM. Illustrative images showing Masson's trichrome staining in 4 *μ*m tissue slides; portraying collagens (blue), muscle fibers, and cell cytoplasm (red) (B). Data are presented as individual animals (mean ± SD,* n* = 5–7) and statistical analysis was performed by the Kruskal–Wallis test with LSD post hoc test; *P ≤ 0.05, **P ≤ 0.01.

In addition, bleomycin administrations caused increased total collagen content in the lung parenchyma (*P* < 0.01), however, treatment with receptor antagonists did not affect the amount of collagen (Fig. 6A and B).

**Figure 7:**
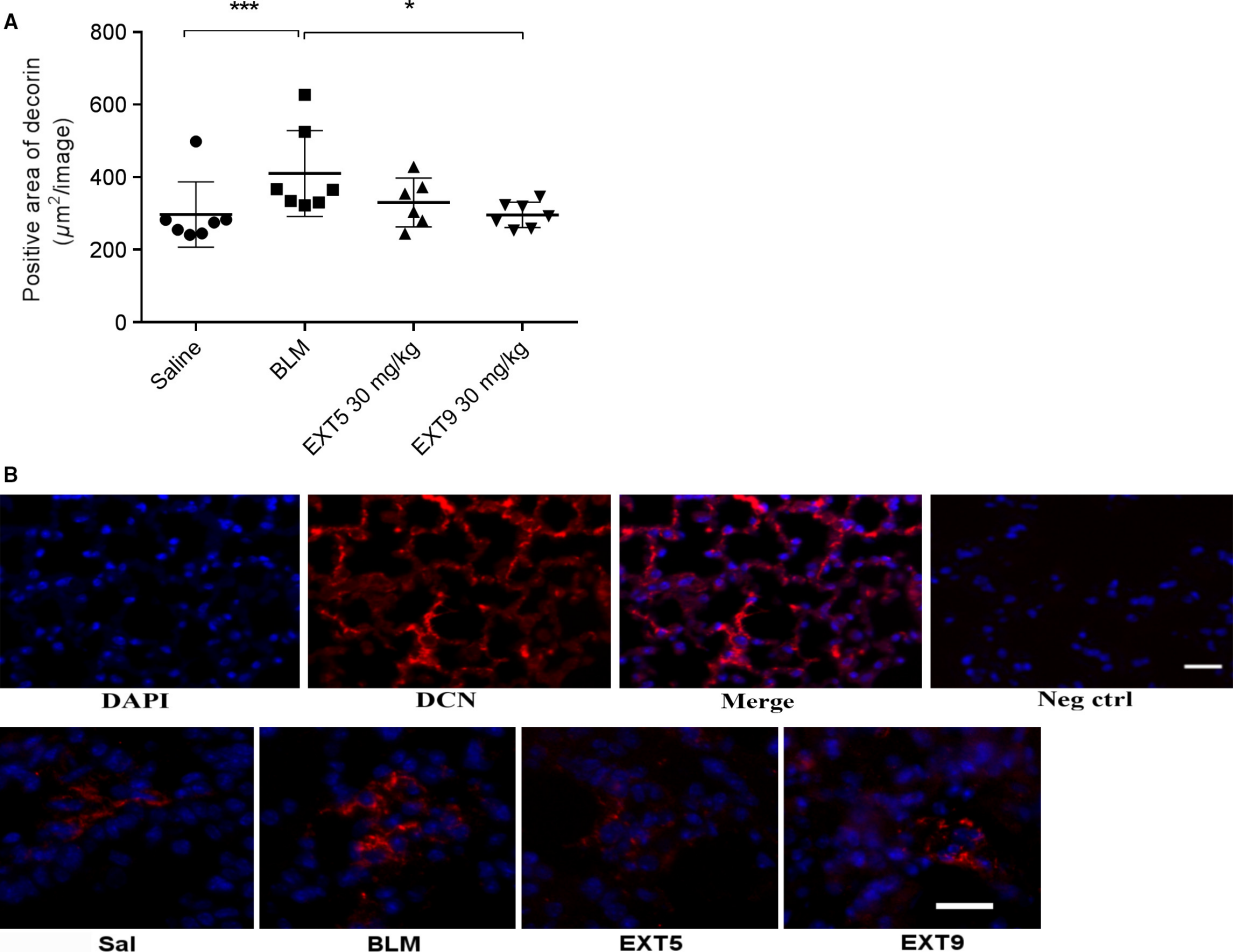
Decorin was increased by bleomycin administration and abolished by 5‐HT_2B_ antagonist. Quantification of positively labeled area showed that bleomycin (BLM) administration increased decorin in lung parenchyma compared to saline (Sal). EXT9 (30 mg/kg) inhibited the BLM‐induced increase in decorin (A). Decorin (red) was detected within the lung parenchyma, nuclei were visualized by DAPI (B). Immunofluorescence negative control (omitting the primary antibody) showed no labeling. Scale bars represent 20 *μ*m and applicable to all images. Data are presented as individual animals (mean ± SD,* n* = 5–7) and statistical analysis was performed by the Kruskal–Wallis test with LSD post hoc test; *P ≤ 0.05, **P ≤ 0.01, ***P ≤ 0.005, ****P ≤ 0.001.

Bleomycin was also associated with a significantly increased decorin expression within the lung parenchyma compared to saline (*P* < 0.005, Fig. 7A and B). This increase was abolished by daily administration of the 5‐HT_2B_ receptor antagonist EXT9 (*P* < 0.05), whereas EXT5 had no significant effect (Fig. 7A).

### Effect of 5‐HT_2B_ receptor antagonists on TNF‐*α* and IL‐1*β* in vivo

**Figure 8:**
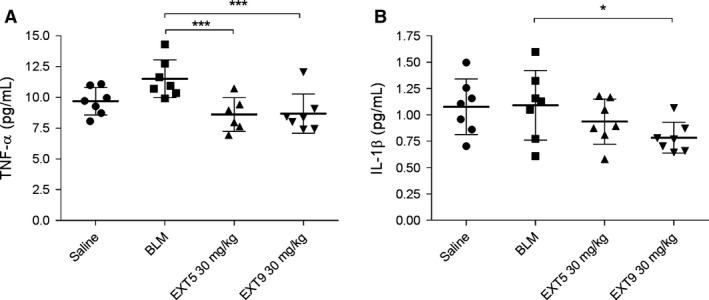
5‐HT_2B_ receptor antagonists decreased serum levels of TNF‐*α* and IL‐1*β*. To investigate the immune response induced by bleomycin (BLM) and the effect of the 5‐HT_2B_ receptor antagonists EXT5 (30 mg/kg) and EXT9 (30 mg/kg), serum was analyzed by a multispot sandwich immunoassay. The level of TNF‐*α* (A) was reduced by the administration of both EXT5 and EXT9, whereas IL‐1*β* (B) was decreased after administration with EXT9. Protein levels are presented as pg/mL, *n* = 6–7, and the Kruskal–Wallis test with LSD post hoc test were used for statistical analysis; *P ≤* *0.05, ***P ≤* *0.005.

Both EXT5 and EXT9 reduced the systemic levels of TNF‐*α* (*P* ≤ 0.005) in comparison to animals receiving bleomycin alone (Fig. 8A). A strong tendency toward bleomycin‐induced increase in TNF‐*α* was found, but we were unable to confirm this statistically (*P* = 0.077). Serum concentration of IL‐1*β* was significantly reduced by EXT9 (*P* = 0.037), compared to animals receiving bleomycin (Fig. 8B). Administration of EXT5 and EXT9 had no significant effect on the production of IL‐2, IL‐4, IL‐5, IL‐6, IL‐10, IL‐12p70, KC‐GRO, or INF*γ*.

## Discussion

The present study highlights a role for 5‐HT_2B_ receptors in the development of pulmonary fibrosis, and our in vitro and in vivo results imply that 5‐HT_2B_ receptor antagonists attenuate myofibroblast differentiation and subsequent ECM synthesis.

Pulmonary fibrosis has been suggested to be the result of aberrant wound healing (Wilson and Wynn 2009), with myofibroblasts considered the key effector cells. Both TGF‐*β*1 and 5‐HT have been shown to induce myofibroblast differentiation and increase ECM deposition in different studies (Lijnen and Petrov 2002; Venkatesan et al. 2002; Konigshoff et al. 2010; Dees et al. 2011). In a different model, where pulmonary fibrosis was induced by intratracheal bleomycin administration, 5‐HT_2A/2B_ receptor antagonist displayed effects on tissue remodeling (Konigshoff et al. 2010). Interestingly, 5‐HT has previously been described to promote tissue repair in liver (Nocito et al. 2007; Ebrahimkhani et al. 2011), highlighting the involvement in regenerative processes.

In this study, biologically relevant concentrations of 5‐HT (Wouters et al. 2007) and TGF‐*β*1 (Blaauboer et al. 2014) (Xu et al. 2007) were used in combination as potent fibrotic stimuli in vitro*,* inducing synthesis of total proteoglycans and the proteoglycan decorin in human lung fibroblasts. 5‐HT alone did not appear to have any significant effects on ECM in our in vitro system; however, other studies have shown that 5‐HT may exert its effect as a helper agonist in synergy with other mediators (Li et al. 1997; Larsson‐Callerfelt et al. 2013a). In similarity to previous studies (Hallgren et al. 2010; Larsson‐Callerfelt et al. 2013b), TGF‐*β*1 did not induce decorin production, which likely is related to the role of decorin as a negative regulator of TGF‐*β*_1_, due to the binding and neutralizing of significant amounts of this growth factor (Yamaguchi et al. 1990). Decorin is also essential for correct collagen fibrillization and thus also deposition (Orgel et al. 2009), and upregulation of decorin has been shown to reduce lung fibrosis induced by TGF‐*β*1 (Kolb et al. 2001). Interestingly, in our in vivo study, subcutaneous bleomycin administration enhanced tissue density, myofibroblast quantity, decorin expression, and collagen deposition and synthesis (measured indirectly as increased collagen‐synthesizing cells by P4H) in the lung parenchyma. Nearly all of these effects were significantly reduced following treatment with the 5‐HT_2B_ receptor antagonists, supporting our obtained in vitro findings in human lung fibroblasts, as shown by a reduction in *α*‐SMA and proteoglycans.

The decreased fibrosis (shown by decreased tissue density in vivo) is probably associated with the reduced numbers of myofibroblasts and collagen‐producing cells. Fewer cells result in decreased decorin synthesis, thus underlining the ability of the 5‐HT_2B_ receptor antagonists to influence the formation of fibrotic tissue. The specific mechanism whereby 5‐HT_2B_ receptor antagonists exert effect on myofibroblast differentiation and potentially ECM production is not well known. Our results support a close interaction between TGF‐*β*1 and 5‐HT, where 5‐HT may potentiate the fibrotic properties of TGF‐*β*1. 5‐HT_2B_ receptor antagonization has been suggested to physically interfere with the TGF‐*β*1 pathway, thus preventing activation of p38 that is essential for myofibroblast differentiation (Hutcheson et al. 2012).

Interestingly, we found that EXT5 and EXT9 displayed somewhat dissimilar effects, both in vivo and in vitro. Our receptor studies indicated that the compounds present high affinity and functionality to the 5‐HT_2B_ receptor and low to moderate affinity to the 5‐HT_2A_ and 5‐HT_2C_ receptors. Thus, brief involvement of these receptors cannot be completely ruled out. The variations of the two compounds in functionality profiles, as well as in binding profiles and possibly also in bioavailability may therefore reveal specific characteristics favorable for regulating diverse antifibrotic events. In this study, we have used a well‐characterized human fetal lung fibroblast cell line that features several advantages in reproducibility and also regenerative capacity; nonetheless primary adult fibroblasts resemble more the clinical picture of lung fibrosis. Further studies with primary cells from patients with different stages of disease are warranted to further explore the role of 5‐HT and effects of 5‐HT_2B_ receptor antagonists.

In this study, we used repeated systemic administrations of bleomycin to induce pulmonary fibrosis (Rydell‐Tormanen et al. 2012; Andersson‐Sjoland et al. 2016), in contrast to most studies that use local (intra‐tracheal) administration. Local administration results in extensive epithelial damage, acute inflammation, and subsequent heterogeneous pulmonary fibrosis. In contrast, systemic administration results in a more homogenous parenchymal fibrosis that develops in parallel with a mild inflammation (Rydell‐Tormanen et al. 2012). The most commonly used approach to study pulmonary fibrosis in vivo relies on homogenization of the lung, thus including pleura, airways, and vessels as well as lung parenchyma, and the possibility to study specific compartments or cell types is rendered impossible. Thus, immunohistochemistry has several advantages in vivo, but most importantly it allows for a detailed study of a specific compartment, such as lung parenchyma that is most commonly affected in pulmonary fibrosis, thus excluding pulmonary vessels and airways. The receptor antagonists EXT5 and EXT9 did not show any significant effect on collagen deposition in the lung parenchyma. However, the evaluation of total collagen quantity does neither consider collagen turnover, subtypes of collagens, nor the structure and stability of the proteins, making it challenging to assess the compounds regulatory effect on this matrix component in relation to the disease model.

**Figure 9:**
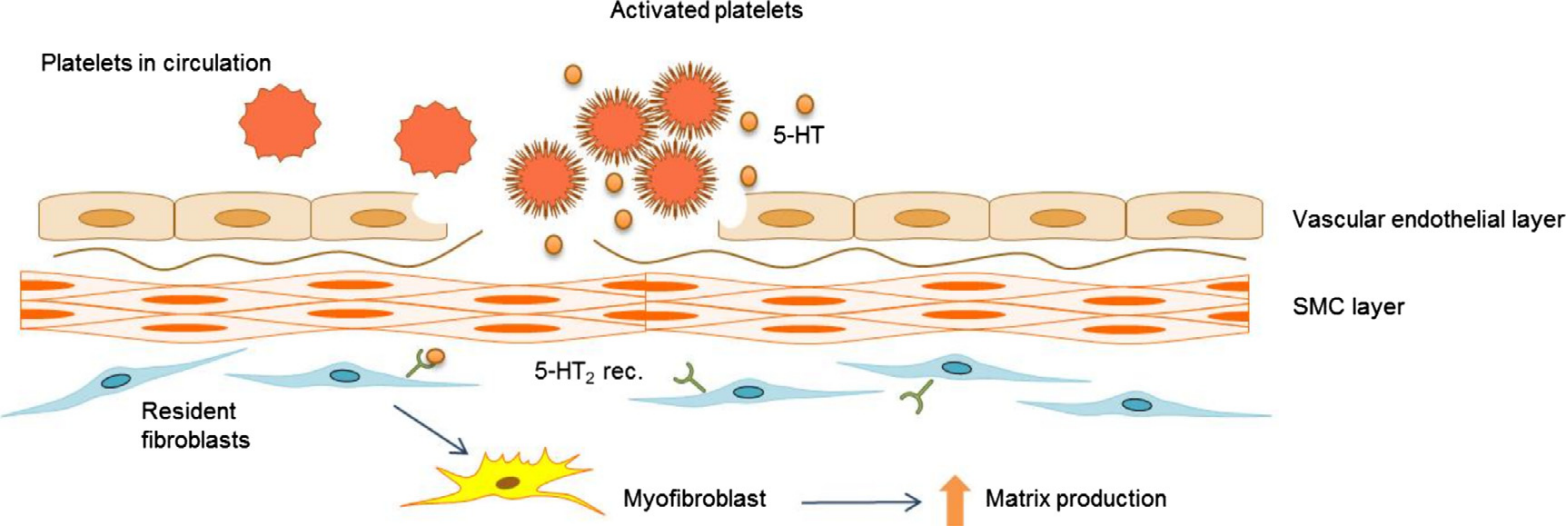
Suggested role of 5‐HT and 5‐HT_2B_ receptors in the development of pulmonary fibrosis. A schematic illustration of a mechanism suggested upon the results obtained in this study. Circulating platelets become activated at sites of vascular injury (e.g., due to endothelial or epithelial damage), and release 5‐HT thus increasing the local concentration of 5‐HT and likely a subsequent release of TGF‐*β*1. 5‐HT binds to 5‐HT_2B_ receptors expressed on resident fibroblast resulting in the progression of myofibroblast differentiation and subsequent increased ECM deposition resulting in fibrotic remodeling. Addition of 5‐HT_2B_ receptor antagonists prevents myofibroblast differentiation and subsequent ECM production.

Pulmonary fibrosis may develop due to diverse pathological conditions (Wynn 2011). However, due to the close proximity between epithelial and endothelial cells within the lung parenchyma, any damage to either cell type will affect the other. By systemic administration of bleomycin, we have emphasized to mimic these processes. Circulating platelets passing a site of activated or injured endothelium will become activated and release 5‐HT, an event that is associated with increased endothelial permeability, myofibroblasts count, and ECM deposition (Dees et al. 2011; Andersson‐Sjoland et al. 2016). The 5‐HT_2B_ receptors have been associated with tissue remodeling caused by platelet activation and vascular damage (Dees et al. 2011), thus highlighting platelet recruitment and activation as potential important fibrotic mediators as hypothesized in Figure 9. In the present study, treatment with 5‐HT_2B_ receptor antagonists also resulted in systemically reduced levels of the proinflammatory cytokine TNF‐*α* and IL‐1*β*, which is supported by previous studies showing anti‐inflammatory properties of these compounds (Wenglén et al. 2010).

Our disease model, utilizing systemic administrations of bleomycin and per oral administrations of therapeutic agents, proposes a systemic evaluation of cytokine regulation. Due to this, broncholavage fluid was not examined as distal pulmonary regions and not central airways were examined. Other studies have shown that systemic administration of bleomycin in mice induces IL‐1*β* in serum, as well as in lung (Hoshino et al. 2009), with similar expression patterns overtime, making the evaluation of serum cytokines essential in examining pathological events in lung fibrosis. This pilot study has generated a first indication of the compounds abilities to regulate cytokine quantities in serum. The study was not optimized for measuring proinflammatory cytokines, for example, time‐dependent sampling, which in part could explain the absence of a significant cytokine increase following bleomycin treatment. The impact on systemic inflammation by these compounds in pulmonary fibrosis warrants further investigation.

## Conclusions

In conclusion, our study implies a profibrotic role for 5‐HT_2B_ receptors in myofibroblast differentiation and ECM production both in vivo and in vitro, and may have an effect on systemic inflammation. We propose these receptors to be important in this regard and further suggest that 5‐HT_2B_ receptor antagonists may have potential to modulate and therapeutic target aspects of profibrotic processes in pulmonary fibrosis.

## Conflict of Interest

A. Löfdahl, C. Wenglén, and G. Ekström were employees of AnaMar AB and own company options at the time of the study. AnaMar AB did not provide support in the form of salaries for authors other than its employees. C. Wenglén and G. Ekström are inventors of patents: UK Priority 1415331.6 filed 29 August 2014 and UK Priority 1511017.4 filed 23 June 2015.
